# High‐resolution climate data reveal an increasing risk of warming‐driven activity restriction for diurnal and nocturnal lizards

**DOI:** 10.1002/ece3.11316

**Published:** 2024-04-29

**Authors:** Pauline C. Dufour, Toby P. N. Tsang, Nicholas Alston, Tristan De Vos, Susana Clusella‐Trullas, Timothy C. Bonebrake

**Affiliations:** ^1^ Area of Biodiversity and Evolution, School of Biological Sciences The University of Hong Kong Hong Kong SAR China; ^2^ Department of Biological Sciences University of Toronto‐Scarborough Toronto Ontario Canada; ^3^ Self‐Employed Cape Town South Africa; ^4^ Department of Botany and Zoology Stellenbosch University Stellenbosch South Africa

**Keywords:** activity patterns, climate change, ectotherms, hours of restriction, intraspecific variation, thermal exposure, warming tolerance

## Abstract

Widespread species experience a variety of climates across their distribution, which can structure their thermal tolerance, and ultimately, responses to climate change. For ectotherms, activity is highly dependent on temperature, its variability and availability of favourable microclimates. Thermal exposure and tolerance may be structured by the availability and heterogeneity of microclimates for species living along temperature and/or precipitation gradients – but patterns and mechanisms underlying such gradients are poorly understood. We measured critical thermal limits (CT_max_ and CT_min_) for five populations of two sympatric lizard species, a nocturnal gecko (*Chondrodactylus bibronii*) and a diurnal skink (*Trachylepis variegata*) and recorded hourly thermal variation for a year in three types of microclimate relevant to the activity of lizards (crevice, full sun and partial shade) for six sites across a precipitation gradient. Using a combination of physiological and modelling approaches, we derived warming tolerance for the present and the end of the century. In the present climate, we found an overall wider thermal tolerance for the nocturnal species relative to the diurnal species, and no variation in CT_max_ but variable CT_min_ along the precipitation gradient for both species. However, warming tolerances varied significantly over the course of the day, across months and microhabitats. The diurnal skink was most restricted in its daily activity in the three driest sites with up to six daily hours of restricted activity in the open (i.e. outside refugia) during the summer months, while the impacts for the nocturnal gecko were less severe, due to its higher CT_max_ and night activity. With climate change, lizards will experience more months where activity is restricted and increased exposure to high temperatures even within the more sheltered microhabitats. Together our results highlight the importance of considering the relevant spatiotemporal scale and habitat for understanding the thermal exposure of diurnal and nocturnal species.

## Graphical abstract

We sampled a diurnal lizard (*Trachylepis variegata*) and a nocturnal lizard (*Chondrodactylus bibronii*) across a diverse climatic gradient in South Africa and measured thermal tolerance and operative temperature data for multiple microhabitats. We found that the diurnal lizard had the most restricted hours of activity during the summer while the nocturnal species was less restricted (being active at night) and exhibited a higher CT_max_. However, under modelled warming scenarios, both species had increased times of restriction, even in sheltered microclimates highlighting the importance of considering multiple temporal and spatial scales in understanding ectotherm vulnerability to warming.

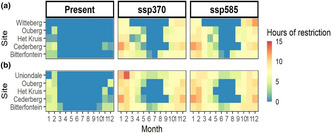

## 1 INTRODUCTION

Ectotherms are sensitive to climate change, as their performance is directly impacted by environmental temperature. Indeed, some lizard species have already undergone local extirpations due to the direct and indirect effects of temperature (Belasen et al., 2017; Sinervo et al., 2010). The thermal sensitivity of lizard performance is bound by upper and lower thermal thresholds, with several sub‐lethal endpoints that define the loss of function (e.g. loss of righting response or onset of spasms at the upper end (the critical thermal maximum, CT_max_) and at the lower end (the critical thermal minimum, CT_min_)) (Angilletta, 2009; Clarke, 2017). A small increase in day versus night temperature and their variability will have different consequences on performance. Daytime warming will bring peak temperatures close to or may even surpass CT_max_, therefore, decreasing performance and forcing animals to either retreat or die. The same small increase in night‐time temperature may result in an increase in performance for nocturnal species but result in a higher resting energetic cost for diurnal species (Rutschmann et al., 2021). Mismatches between the thermal tolerance and the temperature experienced by the organism can restrict vital activities (Belasen et al., 2017; Lara‐Reséndiz et al., 2015). Apart from modifying species distributions, climate change‐driven warming is also expected to modify species behaviour, including restricting activities to shaded habitats and microhabitats or shifting activity to night‐time (Levy et al., 2019).

Variation in critical limits within species can result from adaptation to local climates, different extents of plasticity across populations, degree of genetic isolation, etc. (Chevin et al., 2010). Among populations, variation is, however, essential for assessing the impacts of climate change on species as a whole. Intraspecific differences have frequently been observed in thermal traits, but investigation of projected climate change impacts on these intraspecific differences is rare. For example, Herrando‐Pérez et al. (2019) found that not accounting for intraspecific differences in CT_max_ resulted in both under‐ and over‐estimating impacts of climate change in altering species fitness. Furthermore, for some species, thermal tolerance can vary from day to night (Dufour et al., 2022). Habitat type can also be associated with thermal tolerance variation within species as Landry Yuan et al. (2018) showed that warming benefited ecotone populations but not forest populations of the lizard *Trachylepis affinis*. Similarly, projected impacts of climate change on species behaviour and activity patterns should consider intraspecific differences in thermal parameters to achieve more accurate conclusions.

Here, we used two South African lizard species, one nocturnal gecko and one diurnal skink, to examine how variations in local climates and microclimates shape the opportunities and challenges for activity of lizards in different populations. In this system, we predicted that a precipitation gradient would affect critical temperature limits across populations. According to the Climatic Variability Hypothesis (CVH; Addo‐Bediako et al., 2000; Deutsch et al., 2008; Stevens, 1989), exposure to more variable temperatures may result in wider thermal tolerance ranges to enhance survival (Addo‐Bediako et al., 2000). Dry areas such as deserts tend to have high temporal and spatial thermal variability, with microhabitats offering refuges from extreme temperatures (Belasen et al., 2017). By contrast, wetter areas in temperate zones typically offer lower temperature variability between refuges and open sites, resulting in a narrower thermal tolerance. We hypothesized that microhabitat temperatures, shaped by precipitation‐driven changes in habitat, would influence thermal tolerance, with populations in drier areas exposed to higher variability in temperature and, therefore, having wider thermal tolerance ranges (Clifton & Refsnider, 2022). Further, we expected that the index of warming tolerance (i.e. the amount of buffering from average warming and from extreme high temperatures; Clusella‐Trullas et al., 2021; Deutsch et al., 2008), would be lower in the open (i.e. outside refugia) compared to crevices or refuges in arid areas. Our study, therefore, aimed to quantify intraspecific variation of warming tolerance and activity time of five populations of two widespread lizard species, distributed along a precipitation gradient, under both current and future climatic scenarios. We chose a diurnal and nocturnal species to examine how differences in peak activity time modulate the influence of temperature change on warming tolerance and activity restriction.

## 2 METHODS

### 2.1 Study species

We focused on two widespread, common lizard species, often observed in sympatry: *Chondrodactylus bibronii* and *Trachylepis variegata*. Both species are rupicolous (Bates et al., 2014). *C. bibronii* is also commensal in human habitats, such as barns, old buildings and houses. *T. variegata* can be found under and on bushes (Bates et al., 2014), in sandy gravel areas (Broadley, 2000) and around human construction.

*Chondrodactylus bibronii* (Gekkonidae; SVL = 90 ± 20 mm, Meyer & Mouton, 2007) occurs in the Western and Northern Cape Provinces of South Africa. Despite being primarily nocturnal, they bask near the entrance of their retreat site during the day (Botha, 2010; Patterson, 1987). *Trachylepis variegata* (Scincidae; SVL = 50 ± 7 mm) occurs from western South Africa to southern Namibia (Branch, 1998; Portik, 2009; Portik & Bauer, 2012). *T. variegata* is a diurnal species, emerging as early as 6–7 am until 6–7 pm, but during our sampling period, its activity was reduced during the hottest hours of the day. Nocturnal activity has not been described for this species.

### 2.2 Study sites

**FIGURE 1:**
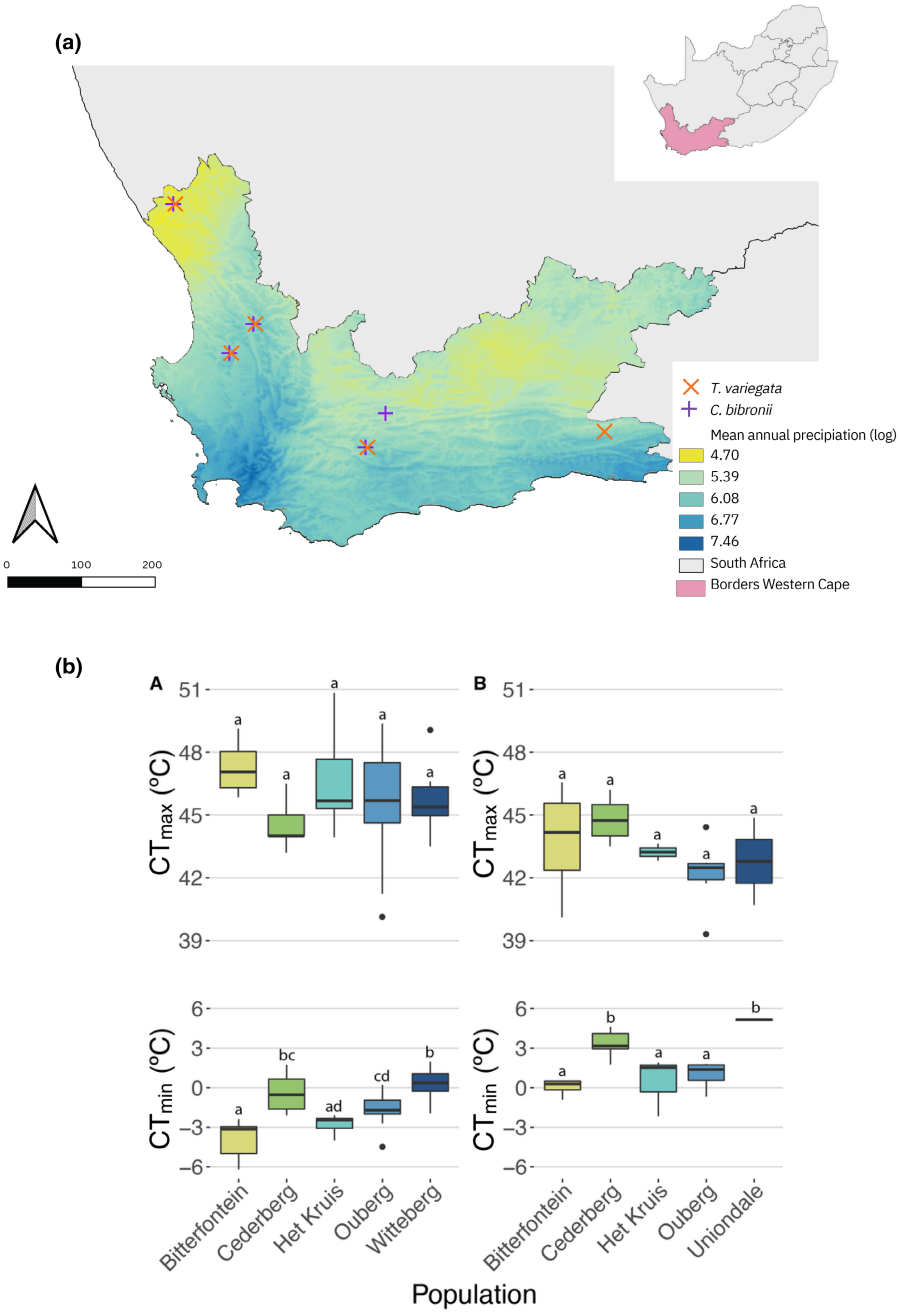
Sampling sites (a) and corresponding thermal tolerances of populations (b) along the precipitation gradient in the Western Cape Province of South Africa. (a) Data on mean annual precipitation from 1981 to 2010 were obtained from CHELSA BIO 12 v 2.1 at 1 km^2^ resolution (Brun et al., 2022; Karger et al., 2017, 2018) and was log‐transformed. Absolute values of mean annual precipitation range from 111 mm (yellow) to 1743 (navy blue) for the whole province. The two species of interest, *T. variegata* (orange cross) and *C. bibronii* (purple cross) were collected from five sites each, of which four overlapped. Map generated with QGIS 3.2. (b) Variation in CT_max_ and CT_min_ for the nocturnal gecko ((A, C), *C. bibronii*, *n* = 43) and the diurnal skink ((B, D), *T. variegata*, *n* = 25) across five sites along a precipitation gradient. Precipitation ranged from 132 mm per year in the driest site (Bitterfontein) to 458 mm in the wettest site (Het Kruis).

Fieldwork took place during two field seasons during the Austral spring, from late September to late November 2018 and 2019, in the Western Cape Province of South Africa. We sampled six different sites differing in precipitation regimes (Table 1; Figure 1). Only one of these sites (i.e. Cederberg) was above 200 m asl (i.e. 230 m asl).

**TABLE 1:** Climate characteristics of the six field sites. Values from CHELSA BIO12, BIO13 and BIO14 downloaded as average from 1970 to 2013.

Site	Coordinates	Year sampled	Mean annual air temperature (min–Max) (°C)	Mean annual precipitation (mm)	Precipitation range driest–wettest month (mm)	Climate (Köppen)
Bitterfontein	−30.99, 18.12	2018	17.45 (11.61–25.19)	132	4–29	Bwh: desert/arid
Witteberg	−33.34, 20.51	2019	15.15 (8.77–22.15)	218	17–40	Bsk: Cold semi‐arid
Ouberg	−33.75, 20.21	2018	16.25 (10.2–22.88)	310	29–43	Bsk: Cold semi‐arid
Cederberg	−32.34, 19.02	2019	18.05 (12.175–24.25)	363	11–122	Csa: hot‐summer Mediterranean
Uniondale	−33.55, 22.94	2019	15.85 (9.76–22.43)	318	20–138	Cfb: Temperate oceanic
Het Kruis	−32.67, 18.75	2018	16.45 (9.91–23.29)	458	10–84	Csa: hot‐summer Mediterranean

The Western Cape Province is unique in terms of diversity of climates, soils and plant assemblages (Goldblatt & Manning, 2002; Manning, 2018). There are two primary precipitation gradients: one permanent, very steep in terms of increased aridity from the south to the north, and a second seasonal gradient from the east to the west, with winter rainfall in the West but increasing summer rainfall in the East. Moreover, the diverse topography creates a rainfall seasonal gradient with altitude. The Cape Fold Belt is a dominant biophysical barrier between the Mediterranean climate to the West and South, and the arid/semi‐arid area that is the Karoo in the North and East. Lizards were captured by hand and/or using a small lasso.

### 2.3 Thermal tolerance assays

After capture, lizards were brought back to the laboratory facilities at Stellenbosch University and were kept for a 7‐day habituation period. Prior to this period, body size measurements were taken (snout–vent length [SVL] and total length) using a calliper and lizards were weighted using a digital balance (Mettler Toledo, New classic MF ML303, ± 0.001 g). They were maintained in individual plastic terraria with substrate and shelter. Light followed a 14D:10N light cycle. They were provided with water daily and food (i.e. two crickets and one mealworm) every other day, as well as the ability to thermoregulate under a heating bulb (up to 30°C on one side of their terrarium between 10 am and 4 pm and room temperature [~24–25°C] at the other end and remaining periods).

Animals were subjected to trials of critical thermal minimum (CT_min_) and maximum (CT_max_) before being released in their natural habitats. Experiments were conducted between 10 am and 4 pm to minimize the potential effect of diel variation (Dufour et al., 2022). Each animal was placed in a small thin metal container, covered by a transparent plastic sheet pierced by small holes to let fresh air in but without risking escape. Each individual was continuously monitored. The container was submerged into a programmable water bath (R4‐GP200, Grant Instruments, Cambridge, UK) and the temperature was kept uniform inside the container. After a 12‐min equilibration period at 25°C, the water was progressively increased (CT_max_) or decreased (CT_min_) by 0.5°C/min (Allen et al., 2016). At every degree above 35°C (CT_max_) or below 10°C (CT_min_), we checked for the loss of righting response defined as the inability of the lizard to right itself when flipped on its back (Brattstrom, 1971; Leal & Gunderson, 2012). To monitor the body temperature, we used a thin thermocouple (type T, 24 SWG, Omega Engineering) inserted inside the container, either at the bottom (skink) or on the side (gecko) to match the preferred resting position of each species. Although we measured the cloacal temperature at the end of each critical limit by inserting a thin thermocouple in the cloaca, we found that temperature of the container was an adequate proxy of body temperature (*R* ^2^ = .89, *p* < .001). After reaching CT_max_ or CT_min_, the animal was directly transferred at room temperature in a moist environment for recovery.

### 2.4 Operative temperature

For ectotherms, behavioural thermoregulation is of primary importance to keep their body temperature within a preferred thermal range, narrower than the available temperatures throughout the environment, and achieve essential tasks such as hunting, fleeing, digesting, mating and resting (Bonebrake et al., 2014; Sunday et al., 2011). To understand the range of environmental temperatures (Te) (or operative temperatures) available for each species, we placed operative temperature models (OTMs) in three types of microhabitats at their place of capture: rock crevices defined here as a crack/fissure on the rock of at least 0.5–1 m in depth? where a lizard had been spotted at the entrance and where direct solar radiation was absent, (“full shade”) partially covered by vegetation (“partial shade”), and on top of rocks and hence fully exposed to direct solar radiation (“full sun”). Each OTM was deployed in pairs to average outlier points but also to increase our chances of retrieval as wildlife or abiotic factors could damage them. The OTM consisted of an iButton logger (thermochron DS1922L, Maxim Integrated) recording temperature every hour over a 12‐month period, suspended inside a thin, flattened, copper pipe (dimensions 7 cm L × 2.5 cm W × 1.2 cm H; 0.5 mm in wall thickness), which was covered with paint that closely matched the range of dorsal skin reflectance of our study species (0.10–0.12; measured with a spectroreflectometer, FieldSpec® 3, ASD Inc., Colorado, USA, range = 350–2500 nm). Each side of the copper pipe was closed using a small cork and adhesive paste. We also recorded hourly air temperature (Ta) at each site using an iButton logger (DS1923L, Maxim Integrated), placed at 1.2 m above ground and inserted at the centre of a white PVC T‐junction to allow convective effects while minimizing direct solar radiation and precipitation.

We deployed OTMs at three sites (Bitterfontein, Ouberg and Het Kruis) in December 2018, and retrieved data every 6 months until November 2019. For the three other sites in Cederberg, Witteberg and Uniondale, we deployed OTMs in October and November 2019 but unfortunately, the COVID‐19 pandemic did not allow us to go back before August and September 2020, reducing data collected in year 2. The Ta logger also failed to record the temperature in Bitterfontein for the first period.

### 2.5 Data analysis

#### 2.5.1 Current

We performed multiple general linear models with CT_max_ or CT_min_ as the response variable, and SVL, population and sex as explanatory variables. We also included the interaction between SVL and population and the interaction between SVL and sex as explanatory variables (both sexes were not captured in every site, so the interaction between sex and population could not be investigated). We analysed each species separately as they occupied different temporal niches. If a variable was significant, we performed a Tukey's posthoc test using the package *emmeans* (Lenth, 2023).

To establish the thermal exposure of each species, we used the index of warming tolerance (WT) (Deutsch et al., 2008). First, we defined WT as the difference between the mean CT_max_ and the mean operative temperature of a population to estimate the relative amount of buffering from warming among populations (Deutsch et al., 2008). We also visualized the diel variation of mean WT (hourly data) for each species, month, site and microsite (crevice as full shade, partial shade and full sun) with a heatmap using the *geom_tile()* function, built in the *ggplot2* package in R (Wickham, 2016). Second, we assessed how activity was restricted by extreme temperatures and how much buffering each population had from extreme temperatures across time. We defined WT as the difference between the population mean CT_max_ and the hourly maximum of environmental temperature (averaged for all days of each month) (e.g. Clusella‐Trullas et al., 2021). We defined the periods and the hours of restriction as the timing and the number of hours per day over which the maximum Te exceeded the lizard's CT_max_ in each microclimate respectively, that is, when WT was lower or equal to 0 (Herrando‐Pérez et al., 2019).

#### 2.5.2 Future

To forecast predictions on the thermal exposure of lizards in the Western Cape in the future and, therefore, to determine how much buffering from temperature extremes would be available to them, we made the following assumptions to simplify our model:

1. Ta will change in the future.
2. The relationships between Ta and Te will remain constant over time.
3. CT_max_ will remain constant over time.

The relationship between Ta and Te is not likely to remain constant over time with variable changes in solar radiation depending on the timing and location (Maclean, 2020). But through this modelling approach, we can better examine the importance of microclimates and tolerance. We retrieved projected temperatures for 2071–2100 from CHELSA: mean, maximum and minimum daily temperature averaged monthly. We chose two climatic GCMs, GFDL‐esm4 (“highest priority” following ISIMIP3b based on CHELSA 2.1. technical specification) and IPSL‐cm6a‐lr and (“second lowest priority” based on CHELSA 2.1 technical specification) representing very different possible climatic outcomes. We extracted Ta, Ta_max and Ta_min in our six field sites, as predicted by IPSL‐cm6a‐lr and GFDL‐esm4, following two of the most likely predicted shared economic pathways (SSP) as described in the sixth assessment of the IPCC: ssp370 and ssp585 (IPCC, 2021). Ssp 370 predicts an increase in global mean annual air temperature of 3.6°C (range 2.8–4.6°C) above the reference value of 1850–1900 and constitutes a medium‐to‐high end of future emissions and warming (O'Neill et al., 2016). Ssp 585 is so far the most extreme with an average increase of 4.4°C (range: 3.3–5.7°C) compared to 1850–1900 (IPCC, 2021).

We also obtained data on maximum daily temperatures averaged by month (Ta max) covering present times (1981–2010) from CHELSA. Note that while the data we collected are from several years after 2010, we felt that this was a better match for “present” time than the 2011–2040 climatology as most of these years have not yet occurred. As our observed data and the projection of current climate are distinct, we needed to calibrate our observed data. We compared maximum Ta max present and Ta max future and defined the “anomaly” (assumption 1) as the difference between the two CHELSA layers Ta future and Ta present.

$$\text{anomaly}=Ta_{\text{model}}\;\text{future}-Ta_{\text{model}}\;\text{present}$$

We then added the anomaly to our observed present‐day data Ta obs to obtain “Ta_obs_ future”.

$$Ta_{\mathrm{obs}}\;\text{future}=Ta_{\mathrm{obs}}\;\text{present}+\text{anomaly}$$

Following assumption 2 (i.e. difference between Ta and Te is constant over time) and based on the equation of “Ta_obs_ future”, we determined “Te_obs_ future” in each microclimate for each site.

$$Te_{\mathrm{obs}}\;\text{future}=Ta_{\mathrm{obs}}\;\text{future}-\left(Ta_{\mathrm{obs}}\;\text{present}-Te_{\mathrm{obs}}\;\text{present}\right)=Te_{\mathrm{obs}}\;\text{present}+\text{anomaly}$$

As CT_max_ does not change (assumption 3) over time, we calculated WT future as the difference between CT_max_ and Te max in the future, for each microhabitat and site.

$$\mathrm{WT}\;\text{future}={\mathrm{CT}}_{\max}-\mathrm{Te}\;\max_{\mathrm{obs}}\;\text{future}$$

## 3 RESULTS

### 3.1 Sampling

We collected 68 individuals (43 geckos and 25 skinks) over six different sites (of which four sites overlap between the two species) (Figure 1, Table 2). Neither mass nor SVL varied significantly between populations for geckos (*p* = .23, *R* ^2^ = .04; *p* = .10; *R* ^2^ = .10 respectively) nor skinks (*p* = .50, *R* ^2^ ~ 0; *p* = .07, *R* ^2^ = .21 respectively).

**TABLE 2:** Morphometric measurements of the two species of interest, *C. bibronii* and *T. variegata*, by population.

Species	Population	SVL (mm)	Total length (mm)	Mass (g)	*N*
*Chondrodactylus bibronii*	Bitterfontein	67.17 ± 18.75	128.39 ± 34.55	19.44 ± 11.81	7
	Cederberg	77.76 ± 4.07	145.76 ± 28.33	15.85 ± 3.53	9
	Het Kruis	68.41 ± 8.98	131.29 ± 27.04	14.5 ± 6.76	8
	Ouberg	71.52 ± 10.88	118.28 ± 22.75	13.21 ± 6.62	9
	Witteberg	79.07 ± 8.33	156.88 ± 20.8	25.58 ± 10.5	10
*Trachylepis variegata*	Bitterfontein	44.61 ± 4.03	85.23 ± 10.91	2.82 ± 1.08	4
	Cederberg	46.81 ± 3.63	94.99 ± 25.32	3.44 ± 0.75	10
	Het Kruis	51.66 ± 4.72	109.74 ± 2.75	4.97 ± 1.3	3
	Ouberg	46.00 ± 4.73	91.92 ± 10.43	5.56 ± 1.3	6
	Uniondale	40.61 ± 0.47	87.34 ± 2.92	1.83 ± 0.02	2

*Note*: Values of SVL (mm), total length (mm) and mass (g) are given as average values ± standard deviation.

### 3.2 Thermal tolerance

Taken together, *SVL*, *population* and their interaction with *sex* explained 48% of the variance observed in CT_max_ of *C. bibronii* (*F*_11,27_ = 2.26; *R* ^2^ = .48; *p* = .041) but none of the variables or interactions was significant (Model 1 in Table S1).

For *C. bibronii*, the model explained 66% of the variation in CT_min_ (*F*_11,27_ = 4.77; *R* ^2^ = .66; *p* < .001). Neither SVL nor sex affected CT_min_ (*p* = .43 and *p* = .95 respectively) but population did (*p* < .0001) (Model 2 in Table S1). Lizards from populations in Witteberg, Ouberg and Cederberg were less tolerant for cold, with predicted 4.4, 2.3 and 4.7°C higher CT_min_ than Bitterfontein (Tukey's posthoc; *p* = .0002; *p* = .04; and *p* = .0011 respectively). Het Kruis had similar CT_min_ than Bitterfontein (Tukey's posthoc; *p* = .41). Lizards from Witteberg had a CT_min_ 2.9°C higher than those in Het Kruis (Tukey's posthoc; *p* = .01).

For *T. variegata*, our model explained 72% of the variance observed (*F*_11,11_ = 2.55; *R* ^2^ = .72; *p* = .06). SVL was not a significant predictor of CT_max_ (Model 3 in Table S2; *p* = .16), but population and sex had a small effect (Model 3 in Table S2; *p* = .04 and *p* = .01 respectively), although less reflected in our posthoc Tukey's test (*p* > .06).

However, for CT_min_, our model explained 81% of the variance observed (Model 4 in Table S1; *F*_11,12_ = 4.57; *p* = .007). Neither SVL nor sex nor the interaction SVL*sex had significant effects on CT_min_ of *T. variegata* (Model 4 in Table S2; *p* = .95; *p* = .36 and *p* = .38 respectively). However, the CT_min_ of lizards from the Cederberg population was 3.01°C higher (i.e. less tolerant for cold) than Bitterfontein's (Tukey's posthoc; *p* = .02), while there was no significant difference between other populations (Tukey's posthoc; *p* > .05).

**FIGURE 2:**
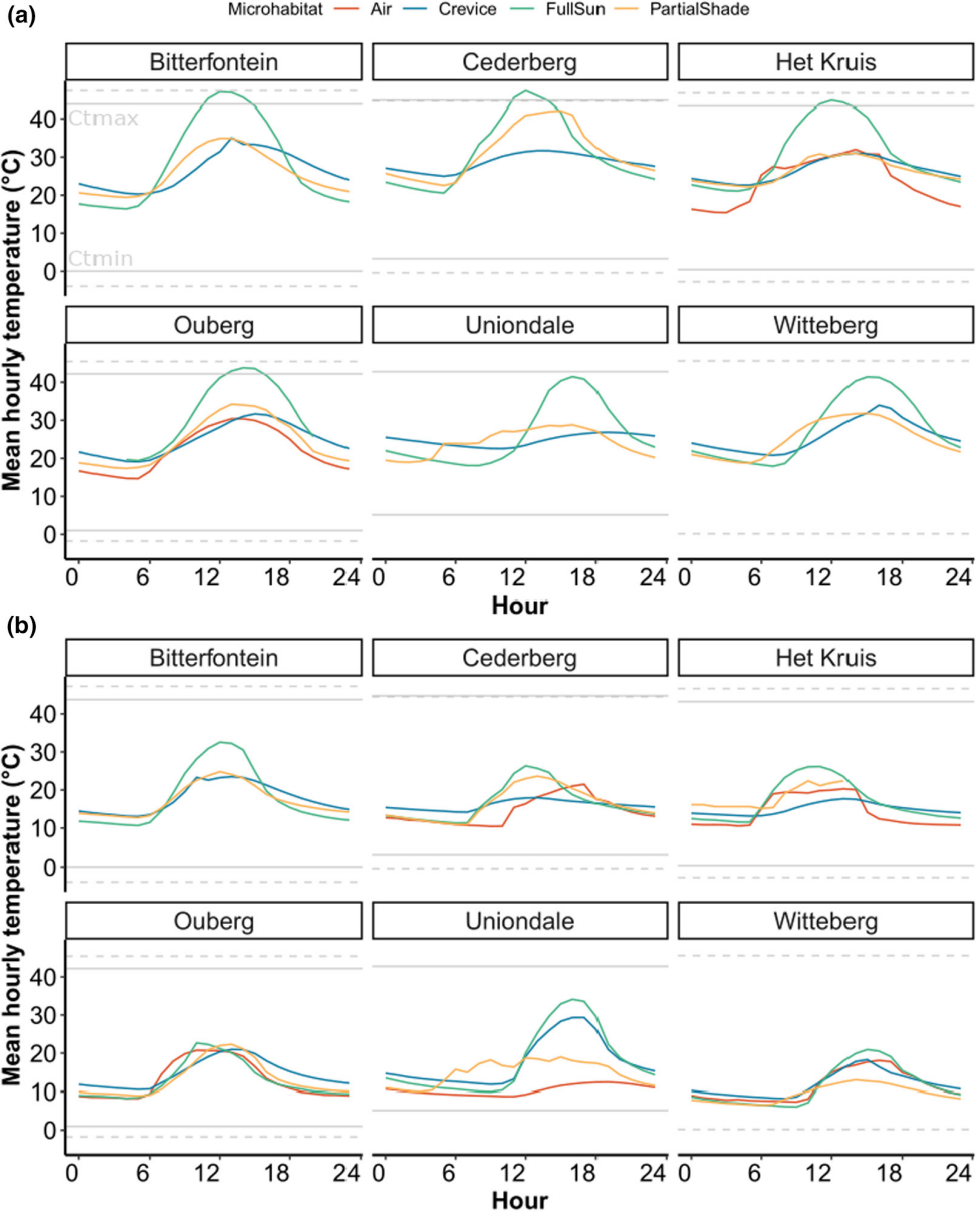
Hourly variation of mean air temperature and mean microhabitat temperature in each of our field sites. Microhabitat included full shade, partial shade and full sun, averaged across the hottest months (a, i.e., December, January and February) and coldest months (b, i.e., June, July and August). Horizontal grey lines represent CT_max_ and CT_min_ of *C. bibronii* (dashed) and *T. variegata* (solid) in each site.

Our field sites experienced different temperature regimes at the microhabitat level (Figure 2). During the hottest months of the year (i.e. December, January and February, Figure 2a), Het Kruis, Uniondale and Witteberg showed the lowest temperature variation across all microsites (crevice, full sun and partial shade), and Bitterfontein, Cederberg and Ouberg the highest. Moreover, operative temperatures in the full sun exceeded CT_max_ of the skink for 2–4 h in Bitterfontein, Het Kruis, and Ouberg, rendering activity on most of the rocks and in partial shade not possible, and the crevices more hospitable microhabitats.

During the coldest months of the year (i.e. June, July and August, Figure 2b), Het Kruis, Ouberg and Cederberg displayed the lowest thermal variation across all three microsites, and Uniondale, Witteberg and Bitterfontein the highest (16.96 ± 8.4°C; 13.5 ± .6.9°C and 17.7 ± 6.9°C respectively). During the winter months (i.e. June, July and August, Figure 2b), neither the mean Ta nor mean Te ever got low enough to approach the CT_min_ of both species.

### 3.3 Buffering thermal extremes and hours of restriction

**FIGURE 3:**
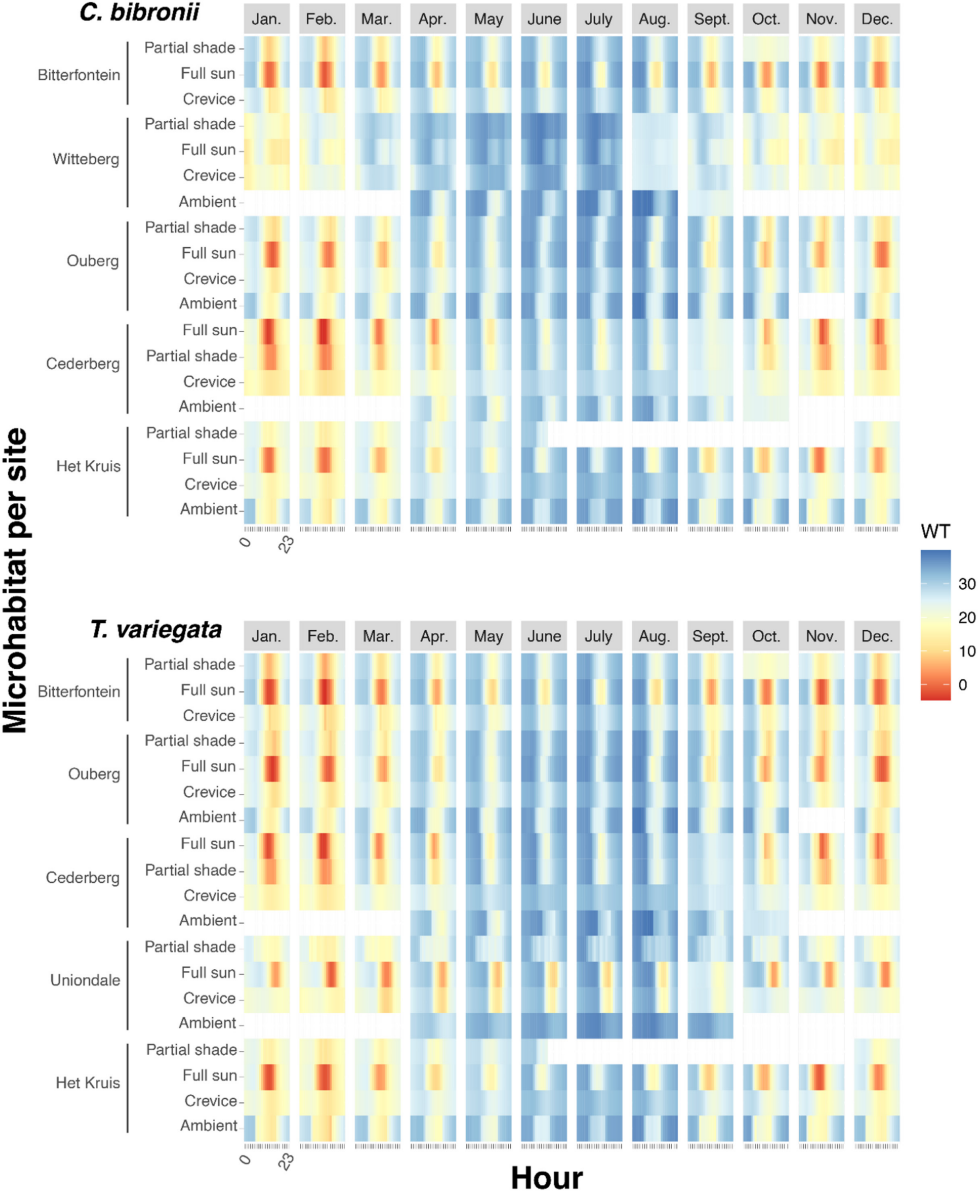
Diel variation of warming tolerance (WT) over the course of 24 h of the nocturnal gecko *C. bibronii* and the diurnal skink *T. variegata*, calculated as mean CT_max_ – mean Te, by population and site, hour and month. WT closer to 0 indicates limited warming tolerance and therefore, less buffer (in red), higher deviations of WT indicate more buffer from warming conditions (in blue).

**FIGURE 4:**
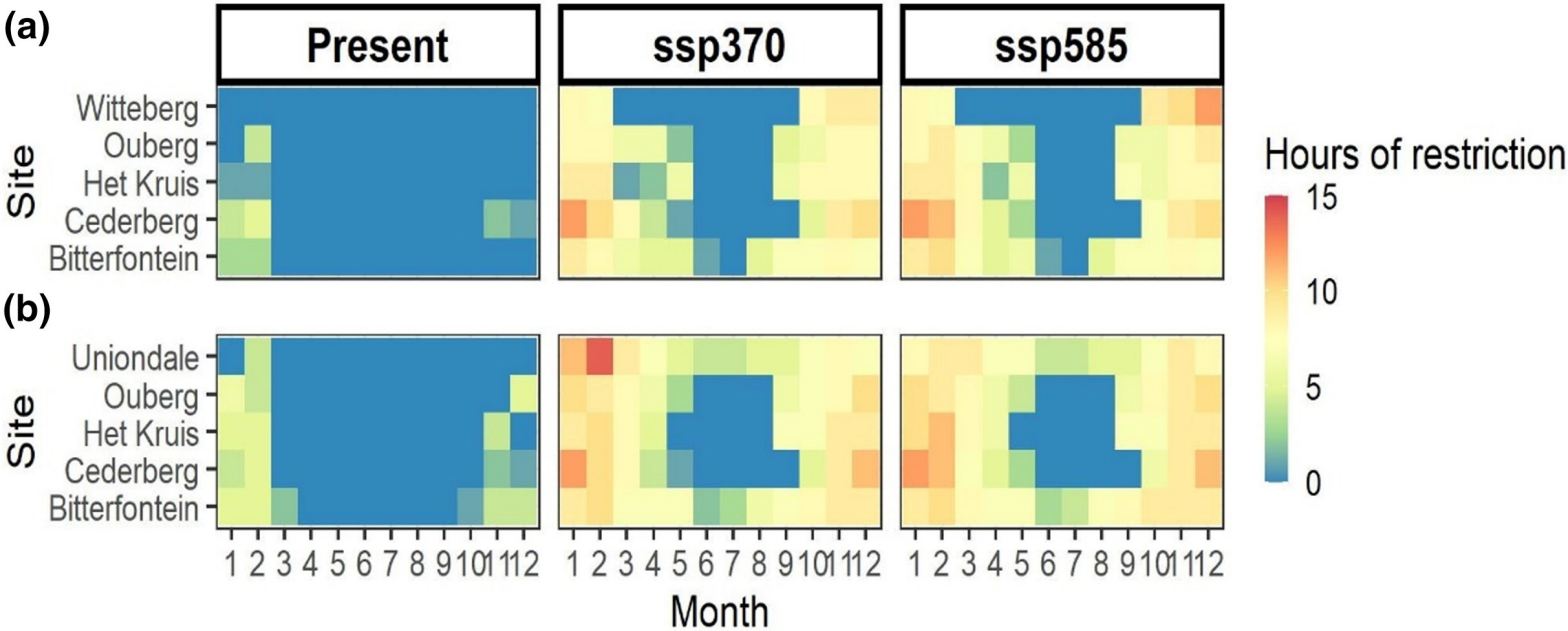
Number of daily hours of restriction of (a) *Chondrodactylus bibronii* and (b) *Trachylepis variegata* in the open (i.e. either full sun and/or partial shade) over the year, in the present and future (scenarios ssp370 and ssp585, averaged between IPSL‐cm6a‐lr and GFDL‐esm4 projections). Activity is considered restricted when WT ≤0.

For this part, we calculated WT as the difference between average CT_max_ of each population minus maximum hourly Te (adapted from Clusella‐Trullas et al., 2021). Given the different temporal niches and heat tolerances of the two study species, high temperatures greatly affected their activity (Figures 3 and 4; Table 3). For the skink *T. variegata*, activity was restricted in the open (i.e. not in refugia, i.e., full sun and partial shade) in all our sites at some point in the year, for as long as 6 h in January in Ouberg (Table 3, Figure 4). In Bitterfontein, hours of restriction occurred 6 months a year and ranged from 1 to 5 h per day (annual average of 1.75 h of restriction per day, Table 3). The night activity of *C. bibronii* was not restricted (no periods above CT_max_ at night‐time nor during early morning/late afternoon) and temperature in the crevices remained below CT_max_ during the hottest hours of the day.

**TABLE 3:** Number of hours of restriction and times of activity restriction in the open (full sun and partial shade) for each month (when WT = mean CT_max_ – max hourly Te ≤0) for each species and site in the present day (i.e. Te observed).

	Jan.	Feb.	Mar.	Oct.	Nov.	Dec.	Annual mean
*C. bibronii*							
Bitterfontein	3 (12–15)	3 (12–15)					0.5
Witteberg							0
Ouberg	4 (13–17)						0.3
Cederberg	3 (11–15)	5 (10–15)			2 (11–13)	1 (12–13)	0.9
Het Kruis	1 (1–2)	1 (1–2)					0.17
*T. variegata*							
Bitterfontein	5 (11–16)	5 (11–16)	2 (13–15)	1 (12–13)	4 (10–14)	4 (11–15)	1.75
Ouberg	6 (12–18)	4 (13–17)				5 (12–19)	1.25
Cederberg	4 (11–15)	5 (10–15)			2 (11–13)	1 (12–13)	1
Uniondale		4 (15–19)					0.33
Het Kruis	5 (11–16)	5 (11–16)			4 (9–13)		1.17

*Note*: Times are given between brackets. Empty cases mean there is no daily temporal restriction. Annual means were calculated as the number of cumulated hours of restriction divided by 12.

Overall, we observed a decrease in WT for both species under the scenarios ssp 370 and ssp 585, in both models – GFDL‐esm4 (Figure S1) and IPSL (Figure S2). Present‐day data indicate that operating in full sun conditions would be equivalent to operating at temperatures exceeding CT_max_ during the hottest months. These temporal restrictions will only be exacerbated with climate change, with normal activity of *T. variegata* restricted, at least partially, during the entire year in Bitterfontein and Uniondale (ranging from an annual average of 7–8.2 h of restriction per day according to the site and scenario; Table S3) between 2071 and 2100 (Figure 4). During the summer months, activity in the open will be restricted for up to 14 h a day for *T. variegata* in Uniondale (Table S3; Figure 4). Moreover, temperatures in the crevices will also be too high at times, rendering the persistence of both *T. variegata* and *C. bibronii* in sites such as Ouberg or Bitterfontein, virtually impossible (Table S3; Figure 4).

## 4 DISCUSSION

We hypothesized that intraspecific variation in microhabitat use and temperature across a precipitation gradient would shape tolerance responses. Along this gradient, we found that different populations had different CT_min_ but similar CT_max_, with wider thermal tolerance (i.e. largest difference between CT_min_ and CT_max_) in the driest and more thermally variable site (Figure 2). Our results, therefore, partially support the CVH in that the most variable site has the widest tolerance but for other sites, there is little consistent variation between variability and tolerance. Warming tolerance, or the extent of available buffer to extreme temperatures, was affected by microhabitat type, with crevices offering the only safe retreat at certain times of the day and during the hottest months of the year in some sites. In the most dramatic case, in the present day, diurnal activity was restricted during 6 months of the year, in the site with the highest CT_max_ (Bitterfontein) which had highly variable temperature between day and night, characteristic of the arid climate of the Succulent Karoo. These trends will only worsen with climate change, making some sites inhospitable for the lizards to persist. Together, our results indicate that the extent of thermal exposure is activity‐, site‐ and microhabitat dependent.

Determining relative sensitivity of different populations of the same species can have important implications for conservation. A consistent CT_max_ but variable CT_min_ among populations aligns with previous work (e.g. Araújo et al., 2013; Leal & Gunderson, 2012; Sinervo et al., 2010) and suggests that the potential for short‐term adaptation to elevated temperatures may be limited (Belasen et al., 2017; Clifton & Refsnider, 2022). Vidan et al. (2017) demonstrated that diurnal lizard species richness could be explained by annual mean temperature (both day and night‐time temperatures) while nocturnal lizards' species richness is driven by night‐time temperatures. Future warming tolerance predictions for this region indicate not only higher daily temperatures but also longer periods (i.e. more hours per day and months per year) of continuous warm temperatures and less refugia, which will have severe consequences on activity periods.

Temporal shifts between diurnal and nocturnal activity have been frequent in lizards (e.g. Gamble et al., 2015) and were also recently documented as a buffering mechanism to elevated temperatures in mammals (Levy et al., 2019). These shifts would have asymmetrical consequences on the performance of diurnal (e.g. Rutschmann et al., 2021) and nocturnal species. To our knowledge, so far, no record of nocturnal activity for *T. variegata* has been described, but with up to six daily hours of restriction during the hottest months of the year in the present and up to 14 h in the future, this species is likely to shift progressively its activity period to be even more matutinal or vespertine. However, the success in catching prey during earlier or later hours of the day is not guaranteed, especially as it can be accompanied by an increased risk of competition with well‐adapted nocturnal species (Crawford, 1931; Pinto et al., 2019) and/or encountering nocturnal predators. Timing mismatches between preferred prey availability and predator requirement can have important consequences for the survival of predators (Durant et al., 2007). In addition, restriction of activity results in a diminution of time spent foraging, mating, and therefore, will negatively affect the fitness of lizards (Sinervo et al., 2010).

By contrast, the nocturnal *C. bibronii* possesses relatively higher tolerance for heat and cold compared to the skink, with a described preferred body temperature comparable to diurnal species (median 31.1°C; *n* = 4; Botha, 2010). Its thermal biology may have resulted from an incomplete shift of geckos to nocturnality (Huey et al., 1989), as *C. bibronii* has also been observed to bask in the morning (Pianka & Huey, 1978). Nocturnal species are predicted to be active at suboptimal temperatures (Huey et al., 1989), and therefore, could benefit from an increase in nocturnal temperatures at least in the short term (e.g. by increasing performance [Angilletta et al., 2010] or expanding their distribution [Vidan et al., 2017]). Elevated temperatures during the resting phase for both diurnal and nocturnal species can also be costly (e.g. shorter resting phase, elevated metabolic rate, higher parasitic loads; Rutschmann et al., 2021). For these lizards, “hotter is not always better” as it signifies shorter activity periods due to increased temperatures but also less and less microhabitats capable of buffering extreme temperatures.

When foraging time decreases drastically, or temperatures become extreme, consequences on physiology can be significant (e.g. Jørgensen et al., 2022), and the likelihood of local lizard extirpations increases dramatically (Sinervo et al., 2010). Dupoué et al. (2017) found that lizard populations exposed to the highest daily temperatures across a mountain range had been undergoing a severe decline in just 12 years, so extirpation of certain populations is possible. Based on our results, *T. variegata* in Uniondale can buffer extreme temperatures more than in Bitterfontein in the present day, but this will no longer be the case at the end of this century (Figure 3). However, our observations of this species in the region around Uniondale were extremely rare, which may indicate either a bias on our part during sampling, or a generally low abundance of this species driven by other factors (predation, low food resources, humidity), despite what temperature suitability may suggest. We know from other studies that humidity at night plays a crucial role in the realized niche of nocturnal geckos (e.g. Puthoff et al., 2010; Vidan et al., 2017) and in the microhabitat selection of both diurnal and nocturnal lizards (Rozen‐Rechels et al., 2019), and therefore, could become a limiting factor as climate becomes drier with climate change in this region (Naik & Abiodun, 2020).

Appropriate spatiotemporal sampling is needed to describe the thermal landscape of ectotherms (Garcia & Clusella‐Trullas, 2019) and microhabitat type (e.g. vegetation and rocks) and their size are particularly important to consider (Stark et al., 2023). Due to constraints of fieldwork, most studies using fine‐scale temperature recording are done on short periods, usually during field seasons (e.g. Brusch et al., 2016; Rutschmann et al., 2020) or focusing on air temperature (e.g. Petford & Alexander, 2021). Meta‐ or modelling studies, on the other end, generate trends at large (regional or global) scale at the cost of the reduction of resolution. We demonstrate here that both temporal (e.g. daily, seasonally or yearly) and spatial (e.g. across microhabitat and site) variations in temperature are important to obtain a more comprehensive assessment of how traits and metrics such as WT of widespread species vary across their distribution range. Average values of thermal traits can also under‐ or over‐estimate the vulnerability of lizards to climate change (Herrando‐Pérez et al., 2019). For instance, by only considering an average CT_min_ in the skink across populations (mean ± SD = 1.97 ± 1.06°C), we would have missed important local variation (ranging from 0.04 ± 0.67°C in Bitterfontein to 5.15 ± 0.13°C in Uniondale). Understanding thermal constraints and opportunities as well as the spatiotemporal scale relevant to a specific group of organisms is of primary importance to adaptative management (Harper et al., 2022). In South Africa, like in many other areas that already experience heat waves and droughts, microclimates can provide significant complexity in habitat heterogeneity. However, microclimatic refugia as a resource are likely to become scarcer and scarcer in a warming world.

## AUTHOR CONTRIBUTIONS

**Pauline C. Dufour:** Conceptualization (lead); data curation (lead); formal analysis (lead); investigation (lead); methodology (lead); project administration (equal; visualization (lead); writing – original draft (lead); writing – review and editing (equal). **Toby P. N. Tsang:** Formal analysis (supporting); methodology (supporting); supervision (supporting); visualization (equal); writing – review and editing (equal). **Nicholas Alston:** Investigation (supporting). **Tristan De Vos:** Investigation (supporting). **Susana Clusella‐Trullas:** Conceptualization (equal); funding acquisition (equal); methodology (equal); project administration (equal); supervision (supporting); writing – review and editing (equal). **Timothy C. Bonebrake:** Conceptualization (equal); funding acquisition (equal); methodology (equal); supervision (lead); writing – review and editing (equal).

## FUNDING INFORMATION

This work was supported by Hong Kong Research Grants Council General Research Fund (17152316) awarded to TCB and SC‐T. Equipment usage in South Africa was supported by South African National Research Foundation funding to SC‐T. PCD was supported by a Hong Kong Postgraduate Fellowship (2017/2021).

## CONFLICT OF INTEREST STATEMENT

The authors declare no conflicts of interest.

## Supporting information


Appendix S1

Data S1

Data S2

## Data Availability

All data are available as files in the Supplementary Material.
