# Supplementary material for: High‐resolution climate data reveal an increasing risk of warming‐driven activity restriction for diurnal and nocturnal lizards
Source: Ecol Evol. 2024 Apr 29;14(5):e11316. doi: 10.1002/ece3.11316 (PMC11056692; doi:10.1002/ece3.11316)
Supplement: Supplementary file 1 — Appendix S1 [file ECE3-14-e11316-s002.docx]

Table S1. Statistical outputs from the Anova Table (II) of the different models used to compare Ctmax and Ctmin across populations and sex and for individuals of different SVL, for *Chondrodactylus bibronii*.

| **Model 1 (*C. bibronii*): Ctmax ~ SVL*sex + population*sex** | | | | |
| --- | --- | --- | --- | --- |
| **Parameter** | **Sum Sq** | **Df** | **F value** | **Pr(>F)** |
| SVL | 8.12 | 1 | 2.46 | 0.13 |
| population | 25.16 | 4 | 1.91 | 0.14 |
| sex | 16.12 | 1 | 4.88 | 0.40 |
| SVL * population | 8.47 | 4 | 0.64 | 0.64 |
| SVL * sex | 4.71 | 1 | 1.43 | 0.24 |
| Residuals | 89.12 | 27 | NA | NA |
| **Model 2 (*C. bibronii*): Ctmin ~ SVL*sex + population*sex** | | | | |
| SVL | 1.13 | 1 | 0.63 | 0.44 |
| population | 62.19 | 4 | 8.61 | 0.00 |
| sex | 0.09 | 1 | 0.05 | 0.83 |
| SVL * population | 7.15 | 4 | 0.99 | 0.43 |
| SVL * sex | 0.32 | 1 | 0.18 | 0.68 |
| Residuals | 48.74 | 27 | NA | NA |

Table S2. Statistical outputs from the Anova Table (II) of the different models used to compare Ctmax and Ctmin across sites and sex and for individuals of different SVL, for *Trachylepis variegata*.

| **Model 3 (*T. variegata*): Ctmax ~ SVL*sex + population*sex** | | | | |
| --- | --- | --- | --- | --- |
| **Parameter** | **Sum Sq** | **Df** | **F value** | **Pr(>F)** |
| SVL | 4.72 | 1 | 2.30 | 0.16 |
| population | 30.71 | 4 | 3.74 | 0.04 |
| sex | 18.37 | 1 | 8.95 | 0.01 |
| SVL * population | 21.80 | 4 | 2.66 | 0.09 |
| SVL * sex | 2.39E-05 | 1 | 1.16E-05 | 1.00 |
| Residuals | 22.57 | 11 | NA | NA |
| **Model 4 (T. variegata): Ctmin ~ SVL*sex + population*sex** | | | | |
| SVL | 0.01 | 1 | 0.00 | 0.95 |
| population | 51.72 | 4 | 9.14 | 0.00 |
| Sex | 1.29 | 1 | 0.91 | 0.36 |
| SVL * population | 4.62 | 4 | 0.82 | 0.54 |
| SVL * sex | 1.17 | 1 | 0.83 | 0.38 |
| Residuals | 16.98 | 12 | NA | NA |


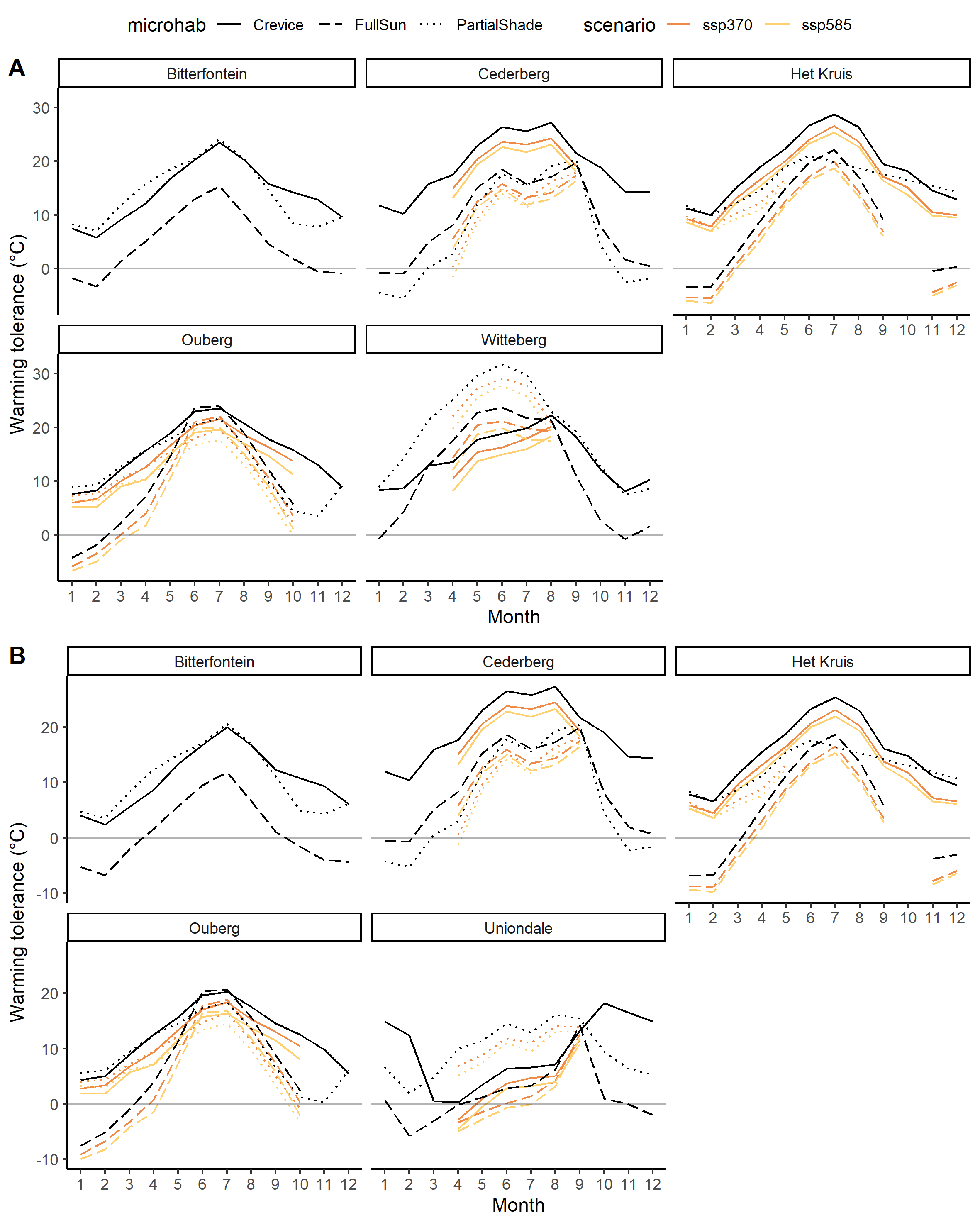


Figure S1. Comparison between present (black) and future (orange and yellow) warming tolerance from extreme temperatures of *Chondrodactylus bibronii* (A) and *Trachylepis variegata* (B) over the course of a year, based on temperature for 2071-2100, predicted by ssp 370 (red) and ssp 585 (yellow) using GFDL-esm4 projection (retrieved from CHELSA, Karger et al. 2017). The black lines represent present day observed data, orange and yellow for ssp370 and ssp585 projected and calibrated data. The grey horizontal line represents the threshold at which WT becomes negative i.e., temperature exceeds the animal’s physiological limits.


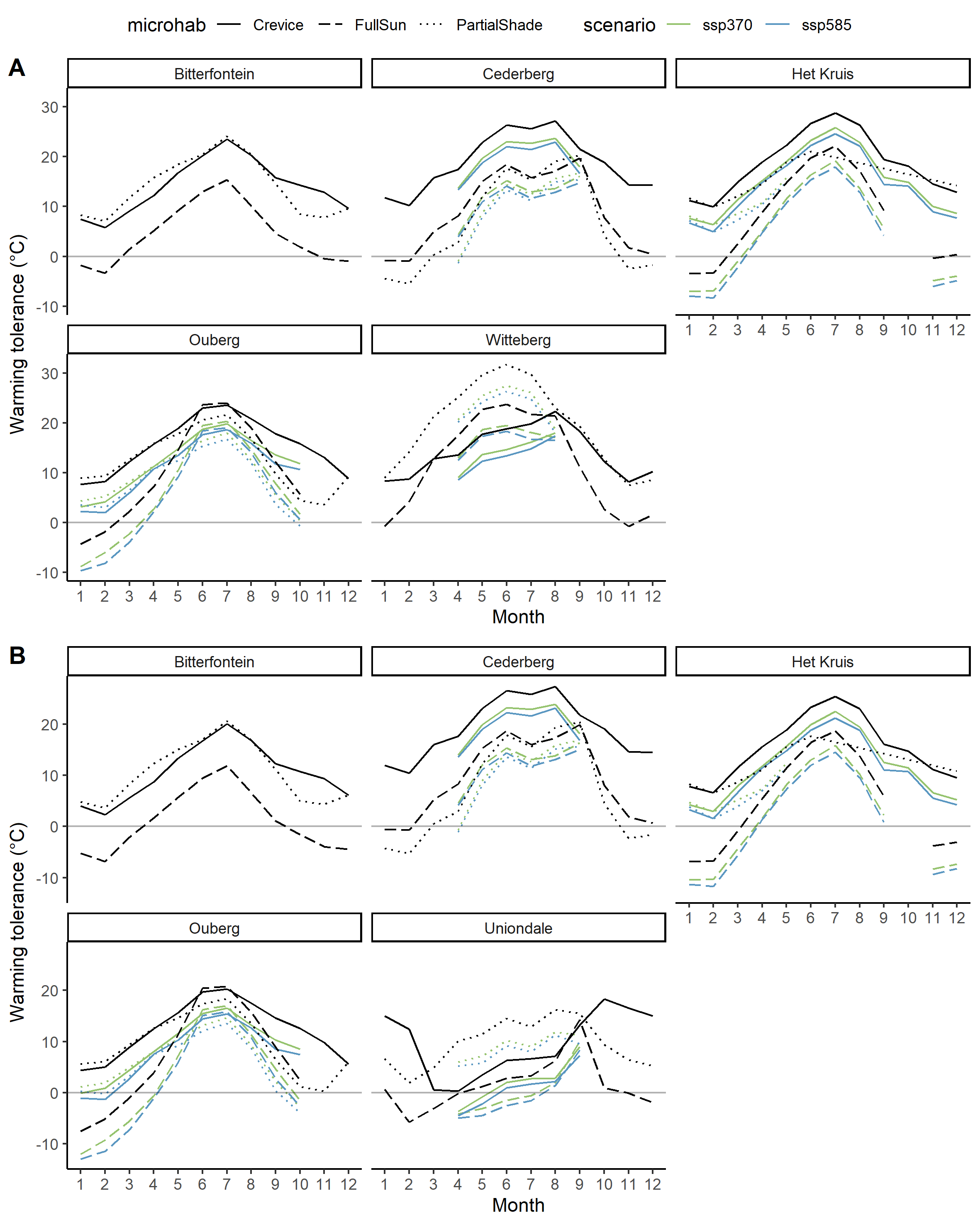


Figure S2. Comparison between present (black) and future (green and blue) warming tolerance of *Chondrodactylus bibronii* (A) and *Trachylepis variegata* (B) over the course of a year, based on temperature for 2071-2100, predicted by ssp 370 (green) and ssp 585 (blue) using IPSL-cm6a-lr projection (retrieved from CHELSA). The black lines represent present day observed data, green and blue represents ssp370 and ssp585 projected and calibrated data. The grey horizontal line represents the threshold at which WT becomes negative i.e., the temperature exceeds the animal’s physiological limits.

Table S3. Mean number of daily hours of restriction and times of activity restriction in the open (full sun and partial shade) per site across the year in the future. Activity is considered restricted when WT (calculated as meanCtmax – max hourly Te) is below 0, and . Two warming scenarios are considered: ssp370 and ssp585. Times are given as HH between brackets. The * signifies that temperature in the crevice at that time is also too hot.

|  |  | **Bitterfontein** | | **Ouberg** | | **Cederberg** | | **Uniondale** | | **Witteberg** | | **Het Kruis** | |
| --- | --- | --- | --- | --- | --- | --- | --- | --- | --- | --- | --- | --- | --- |
|  | **Month** | **ssp370** | **ssp585** | **ssp370** | **ssp585** | **ssp370** | **ssp585** | **ssp370** | **ssp585** | **ssp370** | **ssp585** | **ssp370** | **ssp585** |
| ***T.***  ***variegata*** | Jan | 9 (9-18)* | 9 (9-18)* | 10 (10-20)* | 10 (10-20)* | 12 (7-19) | 12 (7-19) | 11 (10-21) | 8 (13-21) |  |  | 9 (8-17)* | 10 (8-18)* |
|  | Feb | 10 (9-19)* | 10 (9-19)* | 9 (11-20)* | 9 (11-20)* | 10 (8-18) | 11 (8-19) | 14 (7-21) | 14 (7-21) |  |  | 10 (8-18)* | 11 (8-19)* |
|  | Mar | 7 (10-17)* | 8 (10-18)* | 8 (11-19)* | 8 (11-19)* | 8 (9-17) | 7 (9-16) | 9 (12-21)* | 9 (12-21)* |  |  | 8 (9-17) | 8 (9-17) |
|  | Apr | 7 (10-17) | 7 (10-17) | 6 (12-18) | 6 (12-18)* | 4 (11-15) | 5 (10-15) | 7 (13-20) | 7 (13-20) |  |  | 5 (11-16) | 6 (10-16) |
|  | May | 7 (10-17) | 7 (10-17) | 3 (13-16) | 4 (13-17) | 1 (12-13) | 3 (11-14) | 5 (14-19) | 7 (13-20) |  |  |  |  |
|  | Jun | 2 (12-14) | 3 (11-14) |  |  |  |  | 4 (15-19) | 4 (15-19) |  |  |  |  |
|  | Jul | 3 (11-14) | 4 (11-15) |  |  |  |  | 4 (15-19) | 4 (15-19) |  |  |  |  |
|  | Aug | 6 (10-16) | 7 (9-16) |  |  |  |  | 5 (14-19) | 5 (14-19) |  |  |  |  |
|  | Sept | 7 (9-16)* | 8 (8-16)* | 6 (9-15) | 6 (9-15)* |  |  | 5 (14-19) | 5 (14-19) |  |  | 7 (7-14) | 7 (7-14) |
|  | Oct | 8 (8-16)* | 9 (8-17)* | 7 (8-15) | 8 (8-16)* | 5 (11-16) | 6 (10-16) | 7 (13-20) | 7 (13-20) |  |  | 7 (7-14) | 7 (7-14)* |
|  | Nov | 9 (8-17)* | 9 (8-17)* | 8 (8-16)* | 9 (9-18)* | 9 (8-17) | 9 (8-17) | 8 (13-21) | 9 (12-21) |  |  | 9 (6-15)* | 9 (6-15)* |
|  | Dec | 9 (9-18)* | 9 (9-18)* | 10 (10-20)* | 10 (10-20)* | 11 (8-19) | 11 (8-19) | 7 (13-20) | 8 (13-21) |  |  | 9 (8-17)* | 9 (8-17)* |
|  | Annual mean | 7 | 8.17 | 5.83 | 6.08 | 5.0 | 5.3 | 7.16 | 7.25 |  |  | 5.3 | 5.5 |
|  |  |  |  |  |  |  |  |  |  |  |  |  |  |
| ***C.***  ***bibronii*** | Jan | 9 (9-18)* | 9 (9-18)* | 8 (11-19)* | 8 (11-19)* | 12 (7-19) | 12 (7-19) |  |  | 8 (12-20)* | 8 (12-20) | 9 (8-17)* | 9 (8-17)* |
|  | Feb | 8 (10-18) | 10 (8-18)* | 8 (11-19)* | 9 (11-20)* | 10 (8-18) | 11 (8-19) |  |  | 7 (13-20)* | 7 (13-20)* | 9 (8-17)* | 9 (8-17)* |
|  | Mar | 6 (11-17) | 7 (10-17) | 6 (12-18) | 7 (12-19)* | 8 (9-17) | 8 (9-17) |  |  |  |  | 7 (10-17) | 8 (9-17) |
|  | Apr | 5 (11-16) | 5 (11-16) | 6 (12-18) | 6 (12-18) | 4 (11-15) | 5 (10-15) |  |  |  |  | 2 (12-14) | 2 (12-14) |
|  | May | 5 (12-17) | 6 (11-17) | 2 (14-16) | 3 (13-16) | 1 (12-13) | 3 (11-14) |  |  |  |  | 6 (8-14) | 6 (8-14) |
|  | Jun | 1 (12-13) | 1 (12-13) |  |  |  |  |  |  |  |  |  |  |
|  | Jul |  |  |  |  |  |  |  |  |  |  |  |  |
|  | Aug | 5 (10-15) | 5 (10-15) |  |  |  |  |  |  |  |  |  |  |
|  | Sept | 7 (9-16) | 7 (9-16) | 5 (10-15) | 6 (9-15) |  |  |  |  |  |  | 6 (8-14) | 7 (7-14) |
|  | Oct | 7 (9-16) | 7 (9-16) | 6 (9-15) | 6 (9-15) | 5 (11-16) | 7 (10-17) |  |  | 8 (12-20)* | 9 (11-20)* | 8 (6-14) | 6 (8-14) |
|  | Nov | 8 (8-16) | 8 (8-16)* | 8 (8-16) | 8 (8-16)* | 9 (8-17) | 9 (8-17) |  |  | 9 (11-20)* | 10 (11-21)* | 8 (9-17) | 8 (9-17)* |
|  | Dec | 7 (10-17) | 7 (10-17) | 8 (11-19)* | 9 (10-19)* | 10 (8-18) | 10 (8-18) |  |  | 12 (8-20)* | 13 (8-21)* | 8 (9-17) | 8 (9-17) |
|  | Annual mean | 5.7 | 6 | 5.3 | 5.2 | 4.9 | 5.4 |  |  | 3.7 | 3.9 | 5.3 | 5.3 |
